# Anti-*Helicobacter pylori*, Anti-Inflammatory, Cytotoxic, and Antioxidant Activities of Mace Extracts from *Myristica fragrans*

**DOI:** 10.1155/2020/7576818

**Published:** 2020-03-29

**Authors:** Naranpraphai Suthisamphat, Bhanuz Dechayont, Pathompong Phuaklee, Onmanee Prajuabjinda, Ratha-Korn Vilaichone, Arunporn Itharat, Khwanchanok Mokmued, Nuntika Prommee

**Affiliations:** ^1^Department of Applied Thai Traditional Medicine, Faculty of Medicine, Thammasat University, Pathum Thani 12120, Thailand; ^2^Gastroenterology Unit, Department of Medicine, Thammasat University, Pathum Thani 12120, Thailand; ^3^Center of Excellence in Applied Thai Traditional Medicine Research, Thammasat University, Pathum Thani 12120, Thailand; ^4^Division of Applied Thai Traditional Medicine, Faculty of Public Health, Naresuan University, Phitsanulok 65000, Thailand

## Abstract

The aril (mace) of *Myristica fragrans*, known as Dok-Chan, is a spice that has long been used for treating stomach discomfort, peptic ulcer, and nausea. It is an ingredient in many remedies in Thai traditional medicine, e.g., Ya-Hom-Thep-Bha-Jit, Ya-Hom-Nao-Wa-Kot, and Ya-That-Bun-Job, which are used to treat dyspepsia and other gastrointestinal tract symptoms. The aqueous and ethanolic extracts of mace were used for all tests. Anti*-H. pylori* activities were determined by the disc diffusion method and agar dilution. Anti-inflammatory activity was determined by the LPS-induced nitric oxide (NO) inhibition in a RAW264.7 cell line, and cytotoxicity was determined against gastric cancer cell lines (Kato III) using the sulphorhodamine B (SRB) assay. The DPPH radical scavenging and ABTS radical cation decolorization assays were used to determine the antioxidant activities. The result found that the ethanolic extract of mace exhibited antimicrobial activity against *H. pylori* ATCC 43504 and six clinical strains with MIC values of 125–250 *μ*g/ml. The aqueous extract MICs against *H. pylori* ATCC reference strain and six clinical strains were 500 *μ*g/ml compared with 0.5 *μ*g/ml for the positive control, clarithromycin. The inhibitory effect of LPS-induced NO release and cytotoxic activity of the ethanolic extract had IC_50_ values of 82.19 *μ*g/ml and 26.06 *μ*g/ml, respectively, and the EC_50_ values for the DPPH and ABTS antioxidant assays were 13.41 *μ*g/ml and 12.44 *μ*g/ml, respectively. The mace extract also had anticancer properties. In conclusion, the ethanolic mace extract had anti-*H. pylori*, anti-inflammatory, antioxidant, and anticancer activities. These data support further preclinical and clinical investigation to see if the mace extract could have a role in treating patients with dyspepsia, peptic ulcers, and possibly gastric cancer.

## 1. Introduction

The common gastrointestinal (GI) conditions include acid reflux, dyspepsia, nausea, vomiting, peptic ulcer, abdominal pain, flatulence, diarrhea, *elicobacter pylori(H. pylori*) infection, and gastrointestinal cancer [1]. Inflammation is the initial immune reaction in response to cells against pathogens, chemicals, and foreign antigens. NO plays a central role in the regulation of cytokines in the acute inflammatory response and cancer [2]. Increased level of cytokines and oxidants also promote chronic inflammation that can cause DNA damage and the development of cancer [3]. In GI patients, *H. pylori* infection causes gastritis and gastric ulcer which predispose to gastric cancer [4–6].

*Myristica fragrans* (MF) is an evergreen tree whose fruit is used to produce mace (aril) and nutmeg. Both are used commonly for treating stomach discomfort, peptic ulcer, flatulence, and anxiety. Mace and nutmeg are components of many remedies in Thai traditional medicine, e.g., Ya-Hom-Thep-Bha-Jit, Ya-Hom-Nao-Wa-Kot, and Ya-That-Bun-Job, which are used to treat gastrointestinal symptoms [7]. Moreover, they are also used for the same properties in ayurvedic, Chinese medicine, and Tibetan traditional medicine [8, 9].

Nutmeg has antioxidant, antimicrobial, antidiabetic, cytotoxic, anti-inflammatory, and antidepressant activities [10–16]. By contrast, there are few data on the properties of mace, which is well known as Dok-Chan in Thailand. Bhamarapravati et al. [17] reported that dihydroguaiaretic acid (DGA) had antimicrobial activity against *H. pylori*. Neolignans and phenolic compounds, present in the methanolic extract of mace, showed inhibitory effects on mediators in the inflammatory pathway and antiproliferative effects [18]. DNA barcoding of *M. fragrans* (mace) was investigated for the identification of *M. malabarica* adulteration [19]. The safety and efficacy of mace powder combined with pelvic floor muscle training were assessed in a placebo-controlled trial in patients with urinary incontinence. Those receiving mace had greater improvement compared to the placebo group [20].

There is no research data on the use of mace for treating GI tract symptoms. We, therefore, examined the anti-*H. pylori* activity, anti-inflammatory activity, cytotoxic activity, and antioxidant activities of mace.

## 2. Materials and Methods

### 2.1. Bacterial Strains, Cell Cultures, Chemicals, and Reagents

*H. pylori* strain (ATCC 43504) and six clinical isolates of *H. pylori*were obtained from human stomach biopsy specimens kindly provided by Dr. Ratha-korn Vilaichone, Faculty of Medicine, Thammasat University, Thailand. Mouse macrophage leukemia-like cell line (RAW 264.7 and ATCC TIB-71™; Catalog No. 302002.) was obtain from Assoc. Prof. Dr. Arunporn Itharat, Faculty of Medicine, Thammasat University, Thailand. Human gastric carcinoma cell lines (Kato III); catalog no. RCB2088) were purchased from RIKEN BRC, Japan. Columbia agar supplemented with 5% sheep's blood was purchased from RPD, Thailand. Brain heart infusion (BHI) and anaeropack were purchased from Difco, USA, and Mitsubishi gas chemical America, USA, respectively. Clarithromycin, dimethylsulfoxide (DMSO), lipopolysaccharide from *E. coli* O55 : B5 (LPS), sulphorhodamine B (SRB), thiazolyl blue tetrazolium bromide (MTT), and butylated hydroxytoluene (BHT) were purchased from Sigma-Aldrich, MO, USA. Roswell Park Memorial Institute (RPMI) 1640, fetal bovine serum (FBS), trypsin-EDTA, and trypan blue were purchased from Gibco BRL, NY, USA. Penicillin-streptomycin (P/S) and phosphate buffer saline (PBS) were purchased from Biochrom, MA, Germany. Analytical grade reagents (e.g., hydrochloric acid and isopropanol) were purchased from Labscan Limited, Bangkok, Thailand.

### 2.2. Plant Materials and Preparation of the Extracts

Dried arils of *Myristica fragrans* (Mace) were purchased from a herbal shop in January 2017 in Nakhon Pathom province, Thailand. Further botanical identification was conducted at the Department of Applied Thai Traditional Medicine, Faculty of Medicine, Thammasat University. A voucher specimen was deposited in the Herbarium of the Southern Center of Traditional Medicine, Faculty of Pharmaceutical Sciences, Prince of Songkla University, Thailand (SKP 121 13 06 01).

Mace was washed, dried, and grounded before extraction. The aril powder was boiled with water for 30 min and filtered using Whatman paper ^#^1 and then put in the freezer and dried by a lyophilizer to obtain the aqueous extract. For maceration, the powder was soaked with 95% EtOH for three days, filtered and evaporated twice, and then soaked three times to obtain the ethanolic extracts. Both aqueous and ethanolic extracts were refrigerated at−20°C before use.

### 2.3. Determination of Anti-*H. pylori* Activity by Disc Diffusion Method and Agar Dilution Technique

*Helicobacter pylori* ATCC 43504 and six isolates from patients were cultured on Columbia agar supplemented with 5% sheep's blood and incubated at 37°C under microaerobic conditions (N_2_, 85%; O_2_, 5%; CO_2_, 10%) using a gas-generating kit. *H. pylori* were subcultured every 3 days in an anaerobic jar.

The agar disc diffusion technique followed the method by Ogata et al. [21]. Filter paper discs (6 mm in diameter) were impregnated of the extracts (conc. 1 mg/disc). Air-dried discs were placed onto the inoculated Columbia 5% sheep blood agar and incubated at 37°C under microaerobic conditions for 3 days. The triplicate inhibition zones (clear zone) were calculated by measuring the diameter (mm). Clarithromycin (conc. 10 *μ*g/ml) was used as the positive control.

The minimum inhibitory concentration (MIC) was determined by the agar dilution method following the Clinical and Laboratory Standards Institute (CLSI) guidelines [22]. The extracts were serially diluted 2-fold in Columbia agar containing 5% sheep's blood and then transferred separately into Petri dishes. The final concentrations of the extracts in the culture medium ranged from 31.25 to 500 *μ*g/ml. A 72 h bacterial colony of *H. pylori* was harvested and suspended in brain heart infusion (BHI). Bacterial suspensions were prepared to approximately 2.0 of the McFarland standard. Bacterial suspension (3 *μ*l) per spot was replicated on each plate followed by incubating at 37°C for 72 h under microaerobic conditions. The MIC was defined as the lowest concentration of extract which resulted in no visible growth.

### 2.4. Determination of Anti-Inflammatory Activity by Nitric Oxide-Inhibitory Effect

Mouse macrophage leukemia-like cells (RAW264.7, ATCC TIB-71 TM) were induced by lipopolysaccharide (LPS) to release inflammatory mediators including nitric oxide. The Griess reagent was used to determine nitrite, which is a stable end product of NO in cell culture supernatants. The inhibitory effect on NO production was evaluated using the method of Dechayont et al. [23]. Cytotoxicity was also determined using the MTT method. The supernatant in 96-well plates was detected at a wavelength of 570 nm. The percentage of inhibition was calculated using the following formula:

(1) $$\begin{array}{c} \%\text{ inhibition}=\left[\frac{\left(\text{A}-\text{C}\right)-\left(\text{B}-\text{D}\right)}{\;\left(\text{A}-\text{C}\right)}\right]\times 100,\\ \text{control}\left(\text{A}\right):\text{LPS}\left(+\right),\text{test sample}\left(-\right),\\ \text{sample}\left(\text{B}\right):\text{LPS}\left(+\right),\text{test sample}\left(+\right),\\ \text{blank}\left(\text{C}\right):\text{LPS}\left(-\right),\text{test sample}\left(-\right),\\ \text{blank}\left(\text{D}\right):\text{LPS}\left(-\right),\text{test sample}\left(+\right). \end{array}$$

IC_50_ values were calculated form the % inhibition of concentrations using the PRISM program.

### 2.5. Determination of Cytotoxic Activity by Sulphorhodamine B (SRB) Assay

Cytotoxic activity was tested using the SRB assay following the method by Dechayont et al. [24]. Gastric cancer cell lines, Kato III, were cultured in the RPMI 1640 medium supplemented with 10% fetal bovine serum (FBS) and 1% penicillin/streptomycin with CO_2_ incubator at 37°C. The monolayer of cell culture in the flask was trypsinized, and the density was provided 5 × 10 3 cells/well for testing. After various concentrations of extracts (1, 10, 50, and 100) μg/ml were seeded into the 96 well-plate, they were then placed in the same condition. The cells were dyed with SRB, and the viability of the cells was assessed at a wavelength of 492 nm. The percentage of inhibition was calculated by the following formula: [(abs. control − abs. sample)/abs. control] × 100.

### 2.6. Determination of Antioxidant Activities by DPPH Radical Scavenging Assay and ABTS Radical Cation Decolorization Assay

DPPH radical scavenging activity was determined according to the modified method by Yamasaki et al. [25]. DPPH solution in absolute ethanol was freshly prepared before use and protected from light. The extracts were prepared in various concentrations (1, 10, 50, and 100 *μ*g/ml). A portion of the sample solution 0.1 ml was mixed with the DPPH solution (ratio 1 : 1) in 96-well plates and protected from light for 30 minutes at room temperature, and the absorbance was measured at 520 nm using a spectrophotometer.

The ABTS radical cation decolorization assay followed the method by Re et al. [26]. The solution was produced by reacting 7 mM ABTS stock solution in distilled water with 2.45 mM potassium persulfate to produce the ABTS^•+^ solution. The extracts were examined by the ABTS assay. A portion of the sample solution 0.02 ml was mixed with ABTS^•+^ solution (ratio 1 : 10) in 96-well plates and kept in the dark. The reaction was carried out for 6 min, and the absorbance was measured at 734 nm. BHT was used as a positive control in both antioxidant assays.

### 2.7. Statistical Analysis

All experiments were carried out in triplicate and presented as mean ± SEM (standard error of the mean).

## 3. Results and Discussion

### 3.1. Preparation of the Extracts

The aqueous extract (WMACE) and ethanolic extract (EMACE) yields were 6.74% and 28.60%, respectively. In Thailand, nutmeg and mace are commonly combined together and used to be ingredients in the folk medicines which are not only oral administrative drugs but also inhalation drugs. The properties of them recorded in Thai transcripts are quite similar to increasing blood circulation and balancing the digestive system [27]. The major compounds (85%) of chemical constituents of nutmeg and mace are volatile oils which is myristicin [28]. Nutmeg was reported in several activities including antioxidant, antimicrobial, antidiabetic, cytotoxic, anti-inflammatory, and antidepressant activities [10–16]. An adverse effect of myristicin in nutmeg on the central nervous system and psychiatric effect which include hallucinations and delusions are the harmful risks and should be made aware [29]. Recently, an overdose of powdered nutmeg was reported in one case study [30]. Although the FDA has no regulations regarding nutmeg, a suggested limit for powdered nutmeg usage is not more than one teaspoon (approx. 5 g) per day [31, 32]. So, the researchers should find other compounds which would also show efficacy in pharmacology activities.

### 3.2. Anti-*H. pylori* Activity by Disc Diffusion Method and Agar Dilution Technique

**Figure 1:**
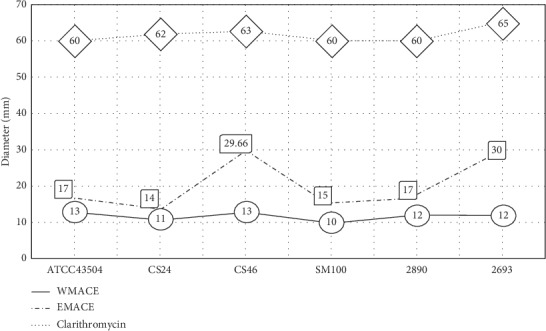
The results of the disc diffusion method of mace extracts and the positive control (clarithromycin) against *H*. *pylori* ATCC reference strain and six clinical strains reported in the clear zone diameter (mm, *n* = 3).

By disc diffusion, the anti-*H. pylori* activity of the mace ethanolic extract, EMACE, was greater than WMACE for the ATCC reference strain and six clinical strains (Figure 1); the MICs for the *H. pylori* ATCC 43504 isolate were 125 vs. 500 *μ*g/ml for EMACE and WMACE, respectively (Table 1). Only the EMACE had activity against the CS24 strain with an MIC value of 250 *μ*g/ml. There are some research studies on chemical constituents and biological activities of mace [33]. Dihydroguaiaretic acid (DGA) was isolated from dried mace which was studied for anti-*H. pylori* activity [17]. The results showed that the diameter of the inhibition zone is 33.7 mm at the concentration 200 *μ*g/ml. Minimum inhibitory concentration (MIC) of DGA against the clinical strains was in range of 100–125 *μ*g/ml [17]. Interestingly, EMACE showed effectiveness exhibited against four clinical strains and ATCC reference strain similar to DGA used in a previous study (MIC values of 125 *μ*g/ml) [17].

### 3.3. Anti-Inflammatory Activity by Nitric Oxide-Inhibitory Effect

For the anti-inflammatory activity vs. LPS-induced NO release, EMACE had an IC_50_ value of 82.19 *μ*g/ml, whereas the MTT assay did not show toxicity to the RAW264.7 cell line for both WMACE and EMACE. In addition, EMACE exhibited activity in the gastric cancer cell lines with an IC_50_ value of 26.06 *μ*g/ml, whereas WMACE had no activity with an MIC >100 *μ*g/ml (Table 2). Previous research has also demonstrated five phenolic compounds which were found from the methanol extract of mace showing inhibitory effects on mediators in the inflammatory pathway and antiproliferative effects. Three compounds showed strong cytotoxic activity in an SRB assay against HT-29 colon cancer cell lines [18]. Moreover, Cuong et al. isolated the phenolic compounds from ethyl acetate soluble fraction of nutmeg. Malabaricone C showed strong inhibitory effect on LPS-induced production of NO in RAW264.7 cells and exhibited the LPS-induced COX-2 and iNOS expressions [15]. In addition, malabaricone C was also found from the organic-soluble extract from *Myristica malabarica* and *Myristica cinnamomea* (Myristaceace family), known for antimicrobial and strong scavenging activities [31, 34]. Banerjee et al. reported a healing activity of malabaricone C against the indomethacin-induced gastric ulceration in mice, and it reduced the ulcer indices better than the positive control, omeprazole with 88.4% and 86.1%, respectively [35]. Other research studies evaluated maceneolignans A, verrucosin, and malabaricone C which were isolated from the methanol extract of mace and inhibited the release of *β*-hexosaminidase in rat basophilic leukemia cells (RBL-2H3) and also inhibited antigen-stimulated tumor necrosis factor-α production [36]. A decoction of pods of *Cassia fistula* Linn. as well as another decoction of arils of *Myristica fragrans* Houtt both significantly reduced pain for a greater length of time than mefenamic acid did in patients with dysmenorrhea. These decoctions both improved patients' quality of life without causing any side effects [37].

### 3.4. Cytotoxic Activity against Gastric Cancer Cell Line (Kato III) by Sulphorhodamine B (SRB) Assay

The results indicated that only EMACE exhibited significant *in vitro* cytotoxic activity against Kato III gastric cancer cells (IC_50_ = 26.06 *μ*g/ml). The WMACE had no significant cytotoxicity (IC_50_ > 100 *μ*g/ml). Notably, several studies found that isolated malabaricone B and C were more effective than regular full-spectrum EMACE whilst some compounds were not [38]. An organic solvent such as methanol, chloroform, and acetone was commonly used for extraction in various studies. None of them extracted the mace based on traditional medicinal usage and demonstrated their GI properties. Thus, our research focused on approving the efficacy of mace for GI treatment in Thai traditional medicinal usage. This is a first report of mace extracts studied on gastric cancer cell lines. As EMACE showed to be effective in all activities, it is possible that the organic solvent can extract an active compound from mace better than water decoction. These reports can support the potential of mace being used as the components of preparation for gastrointestinal symptoms treatment.

### 3.5. Antioxidant Activities by DPPH Radical Scavenging Assay and ABTS Radical Cation Decolorization Assay

**Figure 2:**
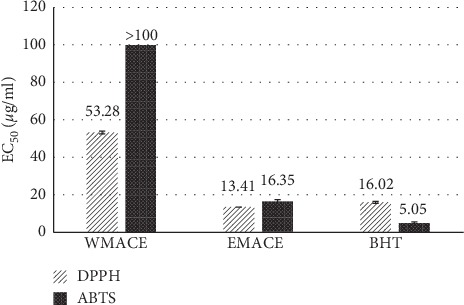
EC_50_ values of the aqueous extract (WMACE) and the ethanolic extract (EMACE) compared with BHT (positive control) in DPPH assay and ABTS assay, *n* = 3.

Antioxidant activities for EMACE and WMACE at concentrations of 100 *μ*g/ml showed similar results with inhibition values of 85.11% and 72.96% in the DPPH radical scavenging assay. The ethanolic extract showed greater antioxidant activities vs. BHT, the positive control, and the EC_50_ values were 13.41 *μ*g/ml and 16.02 *μ*g/ml, respectively. In the ABTS assay, EMACE showed a higher percentage of inhibition at 100 *μ*g/ml, whereas WMACE showed lower than 50%. EC_50_ values are shown in Figure 2. From previous research, the acetone extract of mace exhibited a higher antioxidant activity in the DPPH assay compared to the TLC isolated fractions. On the other hand, all band fractions showed high radical scavenging in ABTS activities [39]. In the same way, the organic extract in our research (EMACE) showed a significant antioxidant activity in DPPH assay and ABTS assay when compare with WMACE.

## 4. Conclusion

The results revealed that the ethanolic extract, EMACE, was dominantly more effective in anti-*H. pylori*, anti-inflammatory, and cytotoxic activities than the aqueous extract, WMACE. Antioxidant activity of EMACE was slightly greater than WMACE. Our research supported the efficacy of using a mace for GI treatment. In future, it would be interesting to study experimentally induced ulcers and cytotoxicity mechanisms and point out the bioactive phytochemicals of mace ethanolic extract of the mace ethanolic extract. According to Thai traditional medicine, mace is a component in the herbal preparations; however, the synergistic effect of the herbal combination in the preparation should be considered.

## Figures and Tables

**Table 1:** The minimum inhibitory concentration (MIC) values of mace extracts and positive control against all *H. pylori* strains (*μ*g/ml, *n* = 3).

Sample	MIC value of *Helicobacter pylori* (*μ*g/ml)					
	ATCC43504	CS24	CS46	SM100	2890	2693
WMACE	500	500	500	500	500	500
EMACE	125	250	125	125	125	125
Clarithromycin	0.5	0.5	0.5	0.5	0.5	0.5

**Table 2:** IC_50_ values of cytotoxic activity against the Kato III cell line and anti-inflammatory activity by inhibiting nitric oxide production in lipopolysaccharide- (LPS-) stimulated RAW 264.7 cells (*μ*g/ml, *n* = 3).

Sample	Cytotoxic activity	Anti-inflammatory activity	
	IC_50_ (*μ*g/ml)	IC_50_ (*μ*g/ml)	% survival
WMACE	>100	>100	113.61
EMACE	26.06 ± 1.95	82.19 ± 4.49	103.66

Cytotoxic activity against the gastric cancer cell line (Kato III) of mace extracts was assessed using the SRB assay. The amount of nitrite in the culture medium was measured by using the Griess reagent, as described in Section 2. Cytotoxicity of RAW 264.7 cell was estimated by the MTT assay.

## Data Availability

The datasets used and/or analyzed during the current study are available from the corresponding author on reasonable request.

## Abbreviations

WMACE: Aqueous extract
EMACE: Ethanolic extract
DGA: Dihydroguaiaretic acid
MIC: Minimum inhibitory concentration
RAW 264.7: Mouse macrophage leukemia-like cell line
BHI: Brain heart infusion
DMSO: Dimethylsulfoxide
LPS: Lipopolysaccharide from *E. coli* O55 : B5
SRB: Sulphorhodamine B
MTT: Thiazolyl blue tetrazolium bromide
BHT: Butylated hydroxytoluene
PBS: Phosphate buffer saline.
